# How Adsorbed Oxygen Atoms Inhibit Hydrogen Dissociation on Tungsten Surfaces

**DOI:** 10.1021/acs.jpclett.2c03684

**Published:** 2023-01-31

**Authors:** A. Rodríguez-Fernández, L. Bonnet, P. Larrégaray, R. Díez Muiño

**Affiliations:** † Université de Bordeaux, ISM, UMR 5255, F-33400 Talence, France; ‡ Centro de Física de Materiales CFM/MPC (CSIC-UPV/EHU), Paseo Manuel de Lardizabal 5, 20018 Donostia-San Sebastián, Spain; § CNRS, ISM, UMR 5255, F-33400 Talence, France; ∥ Donostia International Physics Center (DIPC), Paseo Manuel de Lardizabal 4, 20018 Donostia-San Sebastián, Spain

## Abstract

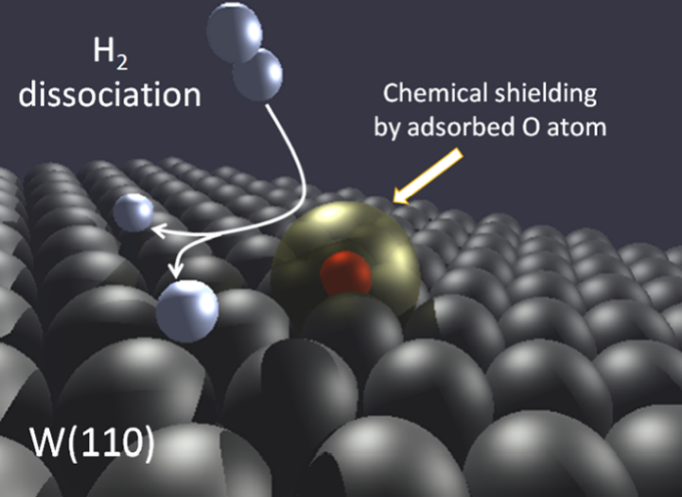

Hydrogen molecules dissociate on clean W(110) surfaces. This reaction is progressively inhibited as the tungsten surface is precovered with oxygen. We use density functional theory and ab initio molecular dynamics to rationalize, at the atomic scale, the influence of the adsorbed O atoms on the H_2_ dissociation process. The reaction probability is calculated for kinetic energies below 300 meV and different O nominal coverages. We show that the adsorbed O atoms act as repulsive centers that modulate the dynamics of the impinging H_2_ molecules by closing dissociation pathways. In agreement with existing experimental information, H_2_ dissociation is absent for an O coverage of half a monolayer. The results show that the influence of O adsorbates on the dissociation dynamics on W(110) goes much beyond the blocking of possible H adsorption sites. Adsorbed O atoms create a sort of chemical shield at the surface that prevents further approach and dissociation of the H_2_ molecules.

Interest in the interaction between hydrogen and metal surfaces has been historically associated with heterogeneous catalysis, the basic route to any large-scale chemical industry production.^1,2^ Hydrogen adsorption on W is one of the simplest chemical reactions that one may envision on a surface. It has been studied for more than 100 years, since Irving Langmuir placed a filament of tungsten in a vessel containing hydrogen gas.^3^ In spite of the extensive coverage of the problem, it is only recently that a basic atomic-level understanding of the mechanisms ruling different reactive processes of hydrogen on W surfaces has been reached.^4−9^ Under well-controlled conditions, the interaction between hydrogen molecules and W surfaces is quite subtle. Small differences in temperature, pressure, or surface termination can drastically affect reactivity. For initial energies below 1 eV and normal incidence, the adsorption probability of H_2_ is much higher on W(100) than on W(110).^10^ This effect is not exclusive of H_2_. For N_2_, the difference between the sticking coefficient on W(100) and on W(110) reaches up to 2 orders of magnitude.^4,11^ The discrepancies between both faces have been explained, for both H_2_ and N_2_, in terms of long-distance interaction and dynamic trapping.^6,7,12^ The interest for H/H_2_ reactivity on tungsten has greatly increased in the past 20 years because of the potential use of tungsten for plasma facing components in nuclear fusion.^13^ The role of oxygen contamination is also a relevant issue as low level of oxygen impurities are usually found in fusion devices that may trigger W surface oxidation,^14^ leading to volatile tungsten oxide^15^ possibly altering the plasma properties.

We recently published a comprehensive theoretical analysis of the dissociative adsorption of H_2_ on W(110) based on ab initio molecular dynamics (AIMD),^9^ relying on density functional theory (DFT) for the electronic degrees of freedom and classical molecular dynamics for nuclei motions. For normal incidence, molecular beam experiments show that there is a monotonical increase of the initial sticking probability *S*_0_ on clean W(110), from *S*_0_ ∼ 0.1 to *S*_0_ ∼ 0.3 when tuning the kinetic energy of the H_2_ beam up to 350 meV.^10^ The theoretical results reproduce reasonably well the experimental data and show that the fate of each trajectory is determined at distances relatively far from the surface (2–2.5 Å).^9^ Only those molecules approaching the surface on top of W atoms can reach the surface and become eventually dissociated.^9^ The necessary accurate description of the long-range interaction between the molecule and the surface therefore requires the inclusion of van der Waals (vdW) terms. After molecular dissociation, H atoms adsorb at the 3-fold coordinated sites of the W(110) surface.^6,8,16^

One of the advantages of AIMD is that the complexity of the system under study can be often increased without much additional computational effort. In the particular case of the current work, we extend the methodology previously used for H_2_ on W(110) to the case of H_2_ impinging on oxidized W surfaces, an intricate problem for which previous studies are scarce. The reliable results previously obtained for the adsorption of H_2_ on W(110) can be used as a reference to quantify the effect induced by the presence of adsorbed oxygen in the H_2_ dissociation dynamics.

The adsorption of oxygen on W(110) is experimentally well characterized. Oxygen molecules dissociate on W(110).^5,17,18^ The very packed nature of the surface does not, however, favor the formation of bulk oxides, which often appear in other W faces. No experimental evidence has been found for oxygen penetration into the bulk through the W(110) face. Isolated oxygen atoms generally adsorb on 3-fold coordinated sites. The 3-fold coordinated sites in a clean W(110) surface are also the most stable ones for hydrogen adsorption. The first effect that oxygen adsorption may have in a later adsorption of H atoms is thus the blocking of possible adsorption sites. Adsorbed oxygen atoms can form well-ordered structures for coverages below one monolayer (1 ML).^5,19,20^ Ordered oxygen islands and disordered domains coexist for some oxygen coverages. Phase transitions can arise as well.^17,21,22^ At room temperature and for 0.5, 0.75, and 1 ML coverages, the structures p(2 × 1), p(2 × 2), and p(1 × 1) are respectively observed.^17,20,23^

There is not much experimental information about the effect that adsorbed oxygen atoms may have in a posterior adsorption of hydrogen on W. Using different experimental techniques, Whitten et al. showed that preadsorbed oxygen decreases the amount of hydrogen that can be adsorbed on W(110).^24^ For coverages of oxygen above 0.35 ML and temperatures of 90 K, they found no significant evidence of H adsorption.^24^ The latter data suggest that the role of adsorbed oxygen goes beyond the possible blocking of adsorption sites for the incoming H atoms. For O coverages below 0.35 ML, the adsorption of H is also clearly below the one found in the clean W(110) surface. Recent studies have also highlight the inhibiting influence of O adatoms on D_2_ chemisorption.^25^

Our purpose in this work is to rationalize the mechanisms that inhibit the dissociative adsorption of hydrogen when oxygen atoms are present at the W(110) surface. The approach is based on dynamical grounds. We focus on three different regimes: (i) the clean W(110) surface, with (ii) 0.25 ML and (iii) 0.5 ML O coverages. Our results show that O atoms adsorbed at the W surface act as repulsive centers that modify the dynamics of the impinging H_2_ molecules. As a result, the surface active sites for H_2_ dissociation are shielded and become inaccessible.

The outline of the paper is as follows. The details of the theoretical procedure and the numerical implementation are described below. They are followed by a presentation of our results as well as a discussion on them. Our conclusions are summarized at the end of the Letter.

A statistically significant number of calculations has been performed for hydrogen molecules impinging on clean and oxidized W(110) surfaces. The coordinate system employed is depicted in Figure 1a. *X*_cm_, *Y*_cm_, and *Z*_cm_ represent the coordinates of the center of mass of the H_2_ molecule while *r*, θ, and ϕ account for the internuclear distance, the polar angle, and the azimuthal angles of the internuclear axis, respectively. Oxygen atoms, when present, are not represented in this panel for clarity purposes. The *Z*_cm_ distance to the surface is always measured relative to the upper layer of W atoms. The unitary cell of the W(110) surface employed in the simulations is shown in Figure 1b. The black circle inside the cell represents an oxygen atom in the 3-fold hollow site. This is the chemisorption position found in various experiments^5,19,23^ for oxygen in the W(110) surface. The chemisorption position is independent of coverage.^5^ All distances are given in units of the W lattice constant *a.*

**Figure 1:**
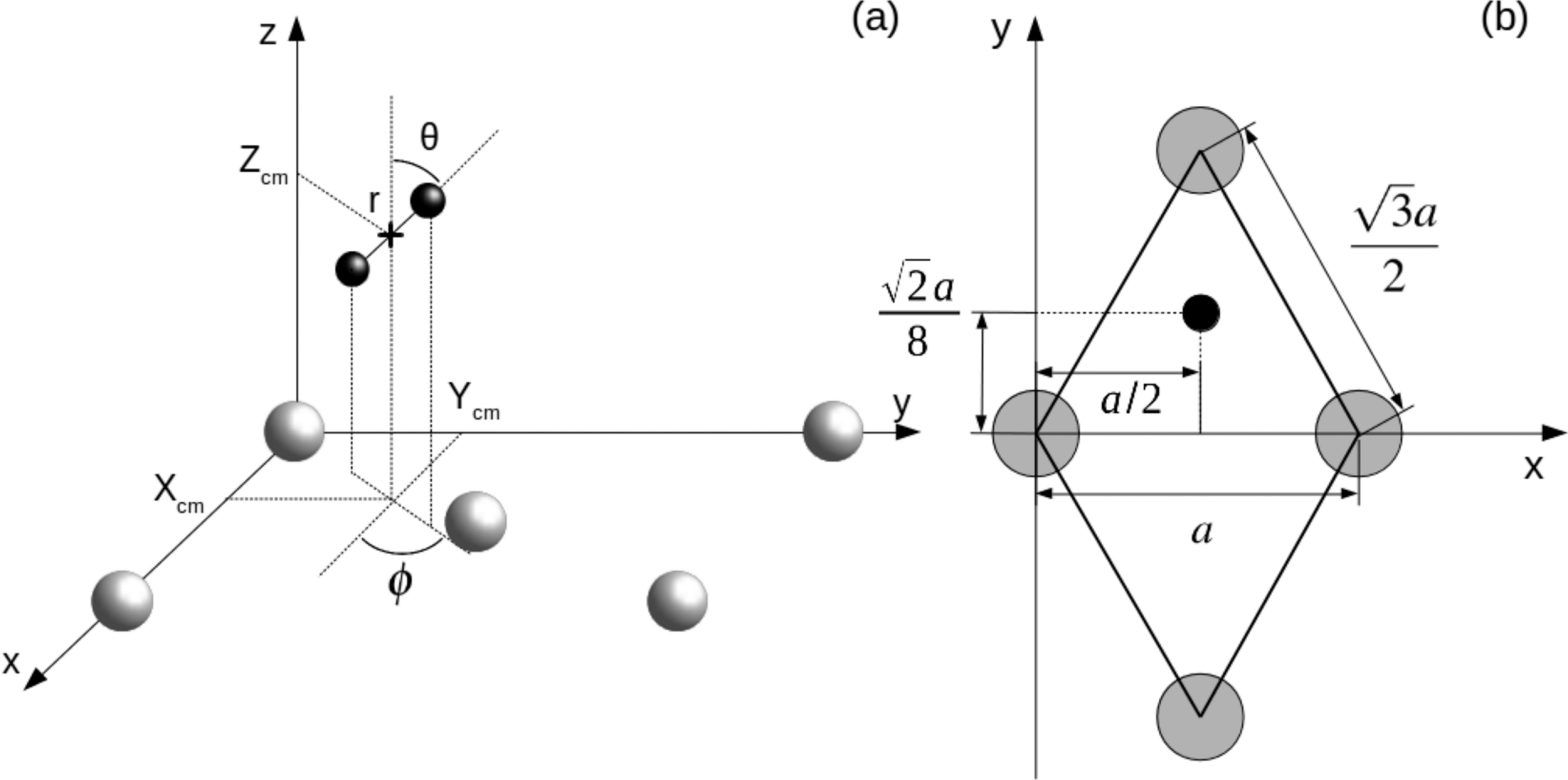
(a) Coordinate system. Gray and black spheres represent W and H atoms, respectively. (b) Surface unit cell of W(110) with an O atom in the stable chemisorption position. Gray circles represent W atoms. The black point represents an oxygen atom in the 3-fold hollow site following results from refs (5, 19, and 23).

Two oxidized surfaces with different coverages have been used in the simulations and are represented in Figure 2. In panel (a) the ordered phase (2 × 1) at coverage Θ = 0.5 monolayers (ML) is shown. Throughout this Letter, Θ stands for coverage in place of the commonly used θ to avoid notation conflicts with the polar angle. Experimentally, the (2 × 1) phase has been thoroughly studied.^5,19,23^

**Figure 2:**
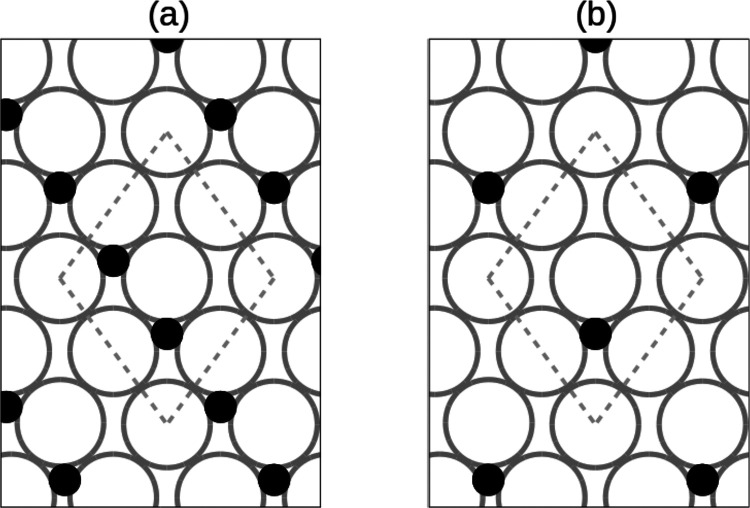
Oxidized surfaces with different coverages. (a) Ordered phase (2 × 1) corresponding to Θ = 0.5 ML. (b) Theoretical model corresponding to Θ = 0.25 ML. Gray and black circles represent W and O atoms, respectively. Dashed gray lines show the boundaries of the cell employed in the calculations.

Experimental results^24^ show that hydrogen adsorption falls to zero for coverages Θ ≥ 0.35 ML. Because of this experimental finding, other ordered phases as (2 × 2) (Θ = 0.75 ML) and (1 × 1) (Θ = 1 ML) present little interest for the present work. For coverages under Θ = 0.5 ML, oxygen islands ordered as the (2 × 1) phase coexist with a disordered array of adsorbed O atoms.^17,23^ In Figure 2b we show a model distribution of O atoms on the surface nominally corresponding to a coverage of Θ = 0.25 ML. This model mimics the situation in which isolated O atoms that do not belong to the (2 × 1) phase can be found at the surface. It will be used to explain what happens for lower O coverages. Tungsten atoms in the corners of the depicted cell do not have any oxygen atoms in the nearby 3-fold sites and will be referred as “isolated”. Therefore, the model for 0.25 ML coverage will represent the situation in which H_2_ molecules may collide with W atoms located at relatively far distances from the O atoms. The size of the cell is small enough to afford a sufficiently large number of AIMD calculations, which are computationally expensive. From this point, the notation Θ = 0.5 ML and Θ = 0.25 ML will be used to refer to the surfaces corresponding to Figure 2.

All the calculations have been performed using the Vienna Ab initio Simulation Package (VASP).^26^ We use pseudopotentials, and only 6 valence electrons are explicitly considered for W and O atoms. The electron–core interaction is treated within the projector augmented wave (PAW) approximation.^27,28^ The exchange-correlation energy is computed using the generalized gradient approximation (GGA) and the vdW xc-functional developed by Dion et al. (vdW-DF).^29^ The implementation in VASP of this functional is performed through the routine written and provided by Klimeš^30^ based on the Román-Pérez and Soler algorithm.^31^

The construction of the slabs used to represent the different surfaces in Figure 2 goes as follows. First, bulk calculations for W are performed obtaining the lattice constant *a* = 3.20 Å. Second, a 5-layer periodic slab of W atoms is relaxed. Each layer in the supercell contains four W atoms as represented with dashed lines in Figure 2. Last, oxygen atom(s) are placed on the 3-fold site(s) at 1.5 Å over the surface, and the system is relaxed again. Using this method, we obtain bond lengths between the O atoms and the nearby W atoms of 2.16 Å, close to the experimental value of 2.08 Å.^19^ This corresponds to a perpendicular distance of 1.21 Å measured from the topmost layer of W atoms in the surface. The supercell dimensions are 5.55 × 5.55 × 27.22 Å^3^, with the first two values being the sides of the rhombus showed in Figure 2 and the last one representing the vertical distance between the first layer of one slab and the first layer of the next one. The vertical distance between two consecutive slabs is approximately 18 Å. These dimensions ensure that the self-interaction of hydrogen is maintained to a minimum while calculation times are kept under reasonable bounds. The total force acting on each atom is kept under 10 meV/Å when the system reaches equilibrium. The energy cutoff used in all the calculations is 400 eV. The fractional occupancies are determined through the broadening approach of Methfessel and Paxton^32^ with *N* = 1 and σ = 0.4 eV. A mesh of 5 × 5 × 1 *k*-points within the Monkhorst–Pack method^33^ is used in all cases. The integration step for all trajectories in AIMD calculations is 2 fs, and the energy convergence threshold for achieving self-consistency is 10^–6^ eV.

Initially, the H_2_ molecules are located above the surface at a distance of 9 Å with the hydrogen atoms separated by the equilibrium distance of 0.741 Å. We consider a grid of initial kinetic energies in the range 50–300 meV, with a 50 meV step. For each energy, 400 trajectories have been simulated at normal incidence. The initial coordinates of the H_2_ molecule in the *XY* plane, as well as the polar θ and azimuthal ϕ angles, are obtained using a conventional Monte Carlo sampling over the whole cell limited by dashed gray lines in Figure 2. The W and O atoms are initially fixed to their equilibrium positions, but they are allowed to move during the dynamics. Energy transfer between the incident molecule and the surface phonons is thus included in the calculations. The zero-point energy of the incident molecule is ignored. The criterion for the dissociation of the H_2_ molecule is established as a separation in the internuclear distance *r* between H atoms of *r* > 1.5 Å and d*r*/d*t* > 0. When the H_2_ molecule is moving away from the surface and the distance between molecule and surface is *Z*_cm_ > 4.5 Å, a reflection event is considered.

Previous calculations of H_2_ impinging on a clean W(110) surface using the same methodology and in the same energy range are available.^9^ Similar parameters have been used in both cases (same supercell dimensions, xc-functional, smearing method, etc.) in order to facilitate the comparison of the results. In particular, the same set of initial coordinates have been used for H_2_ molecules over the clean surface and for Θ = 0.25 ML and Θ = 0.5 ML coverages.

Figure 3 shows the sticking probabilities of H_2_ on W(110) at various oxygen coverages as a function of the collision energy. As already mentioned, experiments^24^ show that for coverages Θ > 0.35 ML hydrogen adsorption gets inhibited by the presence of oxygen. The simulation results are in agreement with this conclusion: at a coverage Θ = 0.5 ML no adsorption is observed in the whole energy range studied. On the other hand, at Θ = 0.25 ML, the sticking coefficient is nonzero and increases with collision energy in a similar fashion as for the clean W(110) surface.^9^ Although not directly comparable, the absolute values of the sticking coefficient seem consistent with the experimental findings: the amount of H adsorbed on W(110) surfaces with 0.25 ML O coverage is roughly 1/4 of the amount of H adsorbed on clean W(110) surfaces.^24^

**Figure 3:**
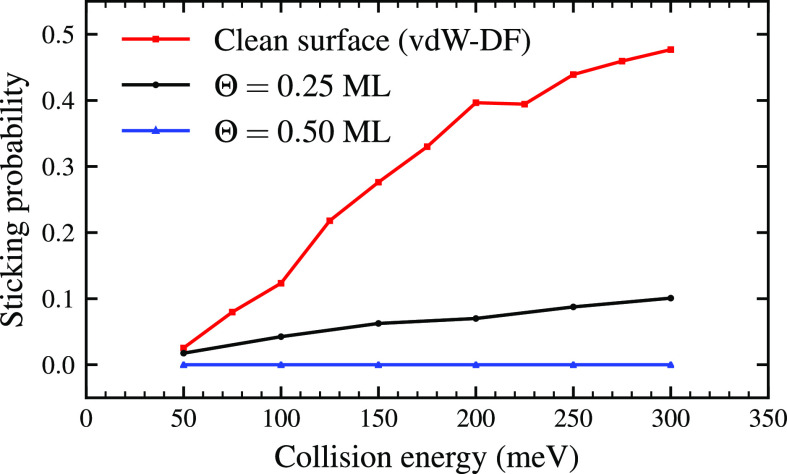
Sticking probabilities as a function of the collision energy. The probabilities drop as the coverage increases for all the collision energies. Similar behavior has been observed experimentally in ref (24).

The time evolution of the *Z*_cm_ coordinate for all trajectories with an initial collision energy of 200 meV is displayed in Figure 4. Different lines represent trajectories with different initial coordinates for a given coverage. The ending of any line before 400 fs corresponds to a dissociation event. Several differences can be spotted for different coverages. First of all, reflection events appear at higher *Z*_cm_ for the oxidized surfaces. The spread of these events also appears to be wider in *Z*_cm_ and in time. For example, for this initial collision energy there is no reflection event with turning point above 2.8 Å in the clean surface. However, for Θ = 0.5 ML there are trajectories that get reflected even at *Z*_cm_ = 3.4 Å. This is a conspicuous effect due to the presence of O repulsive centers above the W atoms.

**Figure 4:**
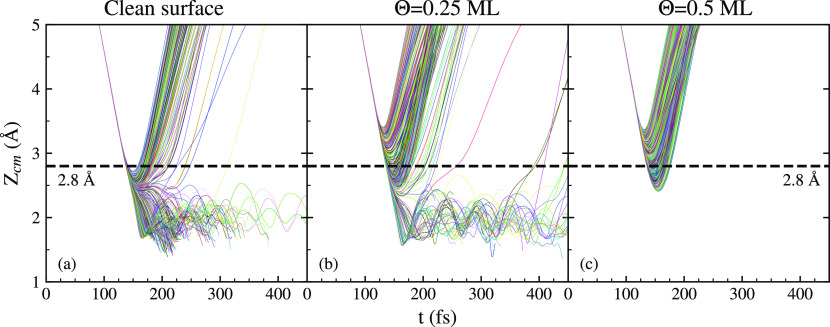
Time evolution of the distance in the *z*-axis between the H_2_ molecule’s center of mass and the surface. Different lines represent trajectories with different initial coordinates for a given coverage. The ending of the lines before 400 fs represents a dissociation event. The initial collision energy is 200 meV in all cases.

The comparison between Figures 4a and 4b shows that the number of trajectories in which molecules are trapped near the surface at 400 fs is much higher in the latter. In a clean surface, only a small percentage of molecules were still trapped at 400 fs and none at 600 fs for all energies studied. Beyond that time, all trapping ended up in dissociation. For Θ = 0.25 ML coverage, the percentage of trajectories trapped for a time longer than 400 fs near the surface increases greatly. These molecules are hovering over the whole cell, and a big part of them end up dissociating and getting adsorbed in the surface. Nonetheless, around ∼20–25% of the trapped molecules at 400 fs go back to the vacuum. This behavior was not observed when a clean surface was used.^9^ Adsorbed oxygen atoms seem to open new paths in the phase space for H_2_ to return to the vacuum. Finally, a small percentage of the total number of molecules are still trapped after 2 ps. The amount depends on the initial kinetic energy, going from 4% at 50 meV to 2% at 150 meV and fully disappearing at 200 meV. For Θ = 0.5 ML, molecules cannot reach the distances to the surface where dissociation occurs for lower coverages (see Figure 4c) . This effectively prevents reactivity. The main features of this figure remain unchanged for different collision energies.

The top panels in Figure 5 show the distribution of the *Z*_cm_ coordinate at the rebound point for all reflected trajectories with initial kinetic energy of 200 meV (same energy as in Figure 4). The bars corresponding to distances under *Z*_cm_ = 2.8 Å have been colored in black. This is the maximum value of *Z*_cm_ for molecules bouncing off a clean surface. Reflection above this point only appears for the oxidized surfaces, and the corresponding bars are colored in red. Bottom panels in Figure 5 show the positions in the *XY* plane of the rebound points for the same trajectories as the top panels. In general, H_2_ molecules reflected at higher distances (in red) correspond to reflections near O atoms. H_2_ molecules reflected at lower distances (in black) interact with W atoms without O atoms nearby. The amount of H_2_ molecules getting reflected above 2.8 Å for Θ = 0.25 ML is roughly half of that for Θ = 0.5 ML. This fact strengthens the conclusion that these rebounds are associated with the presence of O atoms.

**Figure 5:**
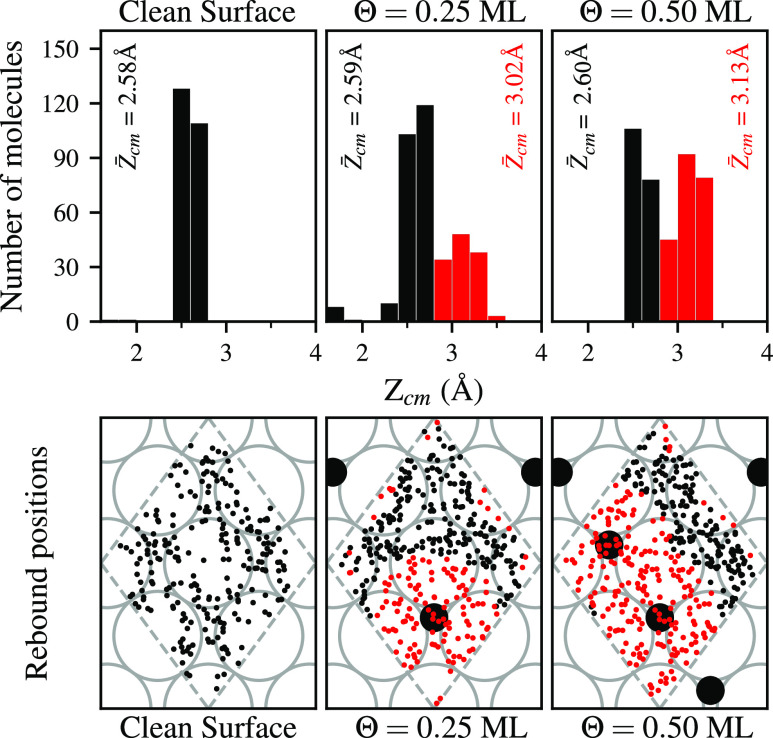
Top panels: distribution of the *Z*_cm_ coordinate at the rebound point for all reflected trajectories with collision energy 200 meV. Bottom panels: positions in the *XY* plane of the rebound points for the same trajectories shown in the top panels. Black and red bars (top) and dots (bottom) respectively correspond to molecules that get reflected on W and O atoms. Gray and black circles respectively stand for W and O atoms in the surface, as in Figure 2. Dashed gray lines show the edges of the supercell in the plane *XY*.

The average of the *Z*_cm_ coordinate for the black and red groups is shown next to the corresponding bars in the top panels of Figure 5. The difference of this value for both groups at the same initial collision energy and coverage is ∼0.4–0.5 Å and does not change with the initial kinetic energy. This value may be perceived as small compared to the vertical distance of O to the first layer of W atoms (∼1.21 Å). The difference in atomic radius between O and W atoms is the main cause of this apparent inconsistency. On the other hand, the average distance to the surface at the rebound points changes with the initial kinetic energy. The higher the energy, the more penetrative power the molecules will have resulting in lower averages of the vertical rebound point. For molecules getting reflected on W atoms (black group) these values are in the range ∼3–2.5 Å corresponding to energies in the range 50–300 meV. Other than this difference in the rebound distance, the general picture shown in Figure 5 is similar for all the range of initial kinetic energies considered.

The reflection process seems very similar for trajectories in the black group no matter the presence of O atoms or not. However, no trajectories lead to H_2_ dissociation at the surface for Θ = 0.5 ML. Molecules do not even get close to the surface according to Figure 4c. Results for H_2_ colliding with a clean W(110) surface^9^ indicate that the H_2_ path to approach the surface and undergo dissociation is narrow. Specifically, only molecules approaching on top of W atoms and with the orientation of the molecular axis roughly parallel to the surface find favorable conditions. Figure 6 shows the position of molecules in the *XY* plane for all simulated trajectories with an initial kinetic energy of 200 meV. The first column of the panels shows the initial position (*t* = 0). The other three panels show the position at later times. Green dots represent molecules following incoming trajectories toward the surface, for distances *Z*_cm_ > 2.5 Å. Blue dots represent molecules following incoming trajectories toward the surface for distances *Z*_cm_ < 2.5 Å. The latter eventually dissociate. Black and red dots represent the trajectories of reflected molecules, i.e., going back to the vacuum. The partition between red and black colors is done in the same way as in Figure 5.

**Figure 6:**
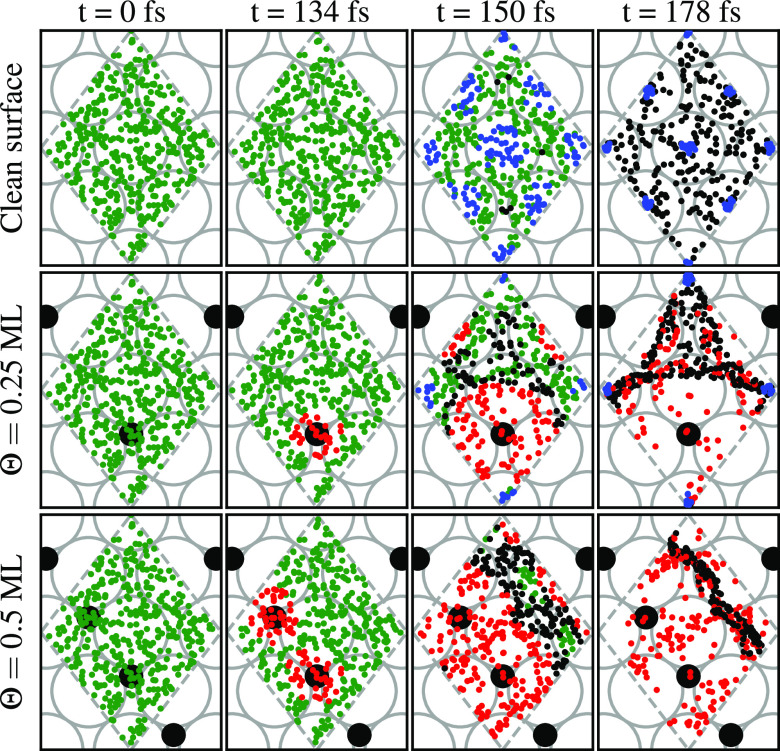
Time evolution of the position of the H_2_ molecules over the *XY* plane. Different times *t* are considered. Gray and black circles symbolize the W and O atoms of the surface. Dots represent the center of mass of molecules over the 2 × 2 cell employed (gray dashed lines). Green dots represent molecules following incoming trajectories toward the surface, for distances *Z*_cm_ > 2.5 Å. Blue dots represent molecules following incoming trajectories toward the surface for distances *Z*_cm_ < 2.5 Å. The latter means that all these trajectories end up in a dissociation process. Black and red dots represent the trajectories of reflected molecules, going back to the vacuum. The initial collision energy is 200 meV for all trajectories. Each row of panels corresponds to different oxygen coverages Θ on the W(110) surface.

In the panels corresponding to the oxidized surfaces, we can observe the strong repulsive effect of the O atoms on H_2_ molecules. The first H_2_ molecules to be reflected are indeed those approaching the surface on the vicinity of the top positions over O atoms. For the rest of the H_2_ molecules getting reflected (in black), a repulsive effect that push them away from the top of the three W atoms closest to O atoms shows up. For Θ = 0.5 ML coverage, this applies to all W atoms. As mentioned above, further approach to the surface would require the H_2_ molecules to be closer to the W top positions. The combination of these two factors helps to explain the lack of molecules approaching shorter distances to the surface.

The situation is slightly different when the coverage is lower. At a coverage of Θ = 0.25 ML one in four W atoms has no O atom in the near 3-fold sites. In the cell, these atoms are located at the four corners. Molecules on top of these atoms do not get pushed away, and the blue dots corresponding to dissociating molecules are exactly on these spots, identical with that of the clean surface. Interestingly, as previously analyzed from Figure 3, dissociation probability is approximately divided by four with respect to the clean surface.

Figure 7 shows the distribution over polar angles θ for trajectories leading to dissociation. The initial kinetic energy is 200 meV. For a clean surface and coverage of Θ = 0.25 ML, there is a predominance in angles close to 90° when the H_2_ molecules go near the surface. All previous results point to the fact that in the zone around the “isolated” W atoms the dynamics are very similar to what was observed for a clean surface.^9^

**Figure 7:**
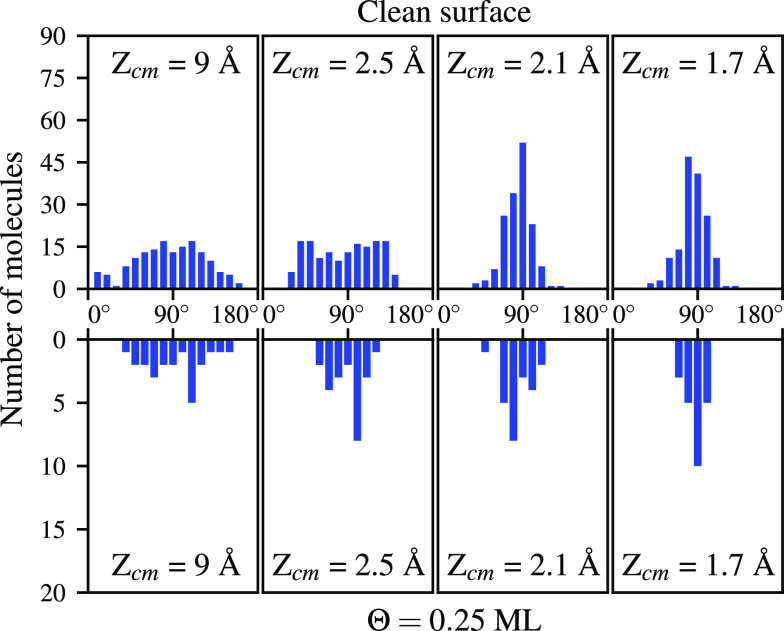
Evolution of the distribution of polar angles θ for the molecules that eventually dissociate at different vertical distances *Z*_cm_. The angular binning is of 10°. Top panels correspond to results on a clean surface and bottom panels to an oxygen coverage of Θ = 0.25 ML. The initial collision energy is 200 meV for all trajectories.

The whole analysis leads to the conclusion that adsorbed oxygen atoms effectively act as repulsive centers at the surface. Each O atom creates in its surroundings an exclusion zone for H_2_ and therefore prevents H_2_ dissociation on the W atoms in its close 3-fold vicinity. Interestingly, from the evolution of H_2_ sticking after prior adsorption of an increasing amount of oxygen, the experiments^24^ suggest that oxygen atoms occupy an effective area 2.85 times larger than that of tungsten atoms. (The effective area of hydrogen atoms is assumed to be the same as the one of tungsten atoms as H coverage reaches 1 ML on clean tungsten.) The present results suggest that oxygen atoms inhibit hydrogen dissociation on the three closest tungsten atoms, thus in nice agreement with experiments.

This idea is confirmed by a parallel analysis in energy terms. Figure 8 shows the interaction energy between H_2_ and the studied surfaces for molecules with initial kinetic energy equal to 200 meV, in the case of a clean surface as well as in the case of surfaces with O coverages of Θ = 0.25 ML and Θ = 0.5 ML. The initial coordinates of the trajectories represented in every panel are the same. We have checked that these trajectories are representative of the general behavior for all kinetic energies considered.

**Figure 8:**
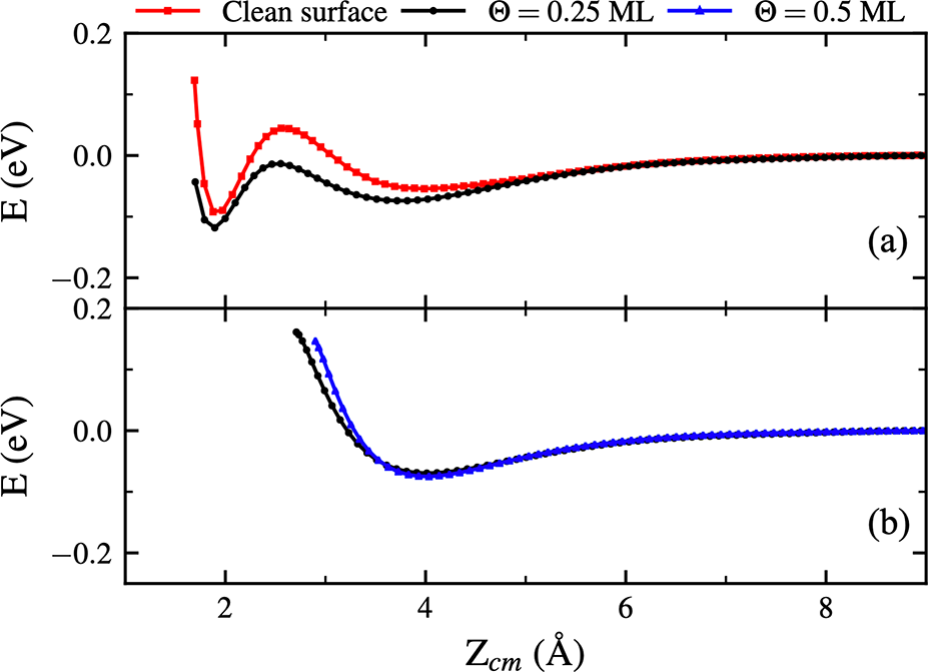
Interaction energy *E* between a H_2_ molecule and the studied surfaces for molecules with initial kinetic energy equal to 200 meV. The lines correspond to a molecule approaching a clean surface (red) and surfaces with Θ = 0.25 ML (black) and Θ = 0.5 ML (blue) O coverages. The initial coordinates for the trajectories inside each of the panels are the same. In the plane *XY*, the initial position is close to (a) the top of W atoms with no O in the nearby 3-fold sites and (b) the top of W atoms with O in one of the nearby 3-fold sites for the oxidized surfaces.

In Figure 8a, the initial position of H_2_ in the *XY* plane is close to the top of W atoms with no O in the near 3-fold sites, which corresponds to all W atoms in a clean surface and 1/4 of the W atoms for Θ = 0.25 ML coverage. These “isolated” W atoms do not exist for Θ = 0.5 ML. Black and red lines are qualitatively very similar, but the presence of O atoms in the surface results in a higher attraction at ∼4 Å and a lower barrier at ∼2.5 Å. The long-range attraction may increase trapping and thus lead some of the molecules to escape back to the vacuum after spending a long time near the surface for Θ = 0.25 ML, as discussed before. In Figure 8a, black and red lines correspond to trajectories leading to adsorption. In Figure 8b, the initial coordinates of H_2_ in the *XY* plane are close to the top of W atoms with O in one of the near 3-fold sites for the oxidized surfaces. At ∼4 Å the oxidized surfaces present a more attractive character, similar to Figure 8a. At closer distances, repulsion by the O atoms switches on. Black and blue lines are very close qualitatively and quantitatively explaining why in Figure 6 adsorption is not observed near this type of W atom.

In summary, we have used AIMD to study the interaction of H_2_ molecules and W(110) surfaces with Θ = 0.25 ML and Θ = 0.5 ML coverages of oxygen atoms. The model we use for the distribution of O atoms at the W surface helps to explain the differences in the dissociative adsorption probability between a clean W(110) surface^9^ and a surface with an oxygen coverage of Θ = 0.5 ML. In the former, only H_2_ molecules on top of W atoms and parallel to the surface will reach distances close enough to undergo dissociation. In the latter, the oxygen atoms push the incident H_2_ molecules away from the top positions, effectively preventing dissociative adsorption at the surface. Therefore, the effect of preadsorbed O atoms at the W(110) surface for H_2_ dissociative adsorption is 2-fold: they block the most stable sites for atomic H adsorption, and they raise additional energy barriers that chemically shield neighboring W atoms against the approach of H_2_.

Qualitatively, our results for the sticking coefficient are consistent with available experimental information based on thermal desorption techniques.^24^ For the case of 0 ML ≤ Θ ≤ 0.35 ML, however, the comparison of our results with experimental data has to be made with caution. For these values of Θ, part of the oxygen atoms may aggregate at the surface and form well-ordered oxygen islands. Statistical kinetic simulations would be necessary on top of the present calculations to describe the full process.

Our calculations on the model surface with oxygen coverage of Θ = 0.25 ML show that the effective influence of the oxygen atoms seems to be restricted to only the closest W atoms. For the rest of W atoms, the dynamics are similar to those for a clean surface. From our calculations we conclude that there are actually two types of W atoms on the oxidized surface: those with an oxygen atom in their vicinity and those with no oxygen atom nearby. The former prevent H_2_ dissociation while the latter provide a path to dissociation in a way similar to the clean surface. This picture may allow to extrapolate the results of our work to other oxygen coverages, provided that phase separation exists.

All in all, we consider that the AIMD calculations presented here provide a very useful ingredient to understand the intricate dynamics of H_2_ when dissociating over such a complex system as the oxidized W(110) surface is.
